# Blood and Bronchoalveolar Lavage Fluid Metagenomic Next-Generation Sequencing in Pneumonia

**DOI:** 10.1155/2020/6839103

**Published:** 2020-08-12

**Authors:** Xu Chen, Shuizi Ding, Cheng Lei, Jieli Qin, Ting Guo, Danhui Yang, Min Yang, Jie Qing, Wenlong He, Min Song, Yan Zhang, Huihui Zeng, Qingwu Qin, Lizhen Yang, Yingjiao Long, Yan Chen, Bingyin Ma, Ruoyun Ouyang, Ping Chen, Hong Luo

**Affiliations:** ^1^Department of Pulmonary and Critical Care Medicine, The Second Xiangya Hospital, Central South University, Changsha, Hunan 410011, China; ^2^Research Unit of Respiratory Disease, Central South University, Changsha, Hunan 410011, China; ^3^Hunan Diagnosis and Treatment Center of Respiratory Disease, Central South University, Changsha, Hunan 410011, China; ^4^BGI Genomics, BGI-Shenzhen, Shenzhen 518083, China; ^5^Tianjin Medical Laboratory, BGI-Tianjin, BGI-Shenzhen, Tianjin, China

## Abstract

**Background:**

Metagenomic next-generation sequencing (mNGS) has made a revolution in the mode of pathogen identification. We decided to explore the diagnostic value of blood and bronchoalveolar lavage fluid (BALF) as mNGS samples in pneumonia.

**Methods:**

We retrospectively reviewed 467 mNGS results and assessed the diagnostic performance of paired blood and BALF mNGS in 39 patients with pneumonia.

**Results:**

For bacteria and fungi, 16 patients had culture-confirmed pathogen diagnosis, while 13 patients were culture-negative. BALF mNGS was more sensitive than blood mNGS (81.3% vs. 25.0%, *p*=0.003), and the specificity in BALF and blood mNGS was not statistically significant different (76.9% vs. 84.6%, *p*=0.317). For 10 patients without culture test, treatments were changed in 2 patients. For viruses, Epstein-Barr virus was positive in blood mNGS in 9 patients. Human adenovirus was detected in both BALF and blood mNGS in 3 patients.

**Conclusion:**

Our study suggests that BALF mNGS is more sensitive than blood mNGS in detecting bacteria and fungi, but blood also has advantages to identify the pathogens of pneumonia, especially for some viruses.

## 1. Introduction

Metagenomic next-generation sequencing (mNGS) has made a revolution in the mode of pathogen identification, as it can detect sequences from all pathogens including bacteria, fungi, viruses, and parasites simultaneously by untargeted sequencing DNA/RNA [1]. Since the reduction of sequencing costs, mNGS has been applied to improve the pathogenic diagnosis in various infectious diseases [2–10]. However, mNGS also has some weaknesses such as high human host background and microbial contaminants that may limit its sensitivity [1]. Compared with the rapid development of mNGS technologies, the lack of clinical studies restricts its clinical application.

Pulmonary infections are the most common infectious diseases. However, the pathogenic diagnoses of pulmonary infections are unclear in nearly half of patients in China, although various conventional pathogen-detecting tests have been conducted [11]. This may be partly because of the frequent use of prior antibiotic therapy before pathogen detecting tests sampling. It has been shown that the yield of conventional culture in pulmonary infections is usually low, especially in those with prior antibiotic therapy [12]. Identification of the etiological pathogen is crucial for targeted antimicrobic therapy and reducing unnecessary antibiotic treatment and is hopeful to help blunt the rapidly increasing antimicrobial drug resistance. A recent study showed that mNGS was less affected by prior antibiotic exposure than conventional culture [8]. Thus, mNGS may be a comprehensive diagnostic tool to aid the pathogenic diagnosis of pulmonary infections [13, 14].

The most common sample types used in conventional bacterial or fungal culture include blood and respiratory tract samples such as bronchoalveolar lavage fluid (BALF) and sputum. Studies have shown that blood culture should not be recommended even in severe pulmonary infections due to its extremely low yield [15, 16]. Contrary to this, molecular diagnostic methods such as polymerase chain reaction (PCR) and mNGS have been proven feasible in blood samples to detect pathogens in pulmonary infections [17, 18]. As an evolutional diagnostic tool, the positive rate of BALF and blood mNGS in pulmonary infections is still unknown. Our study aims to evaluate and compare the diagnostic performance of BALF and blood mNGS to detect pathogens including bacteria, fungi, and viruses in pneumonia.

## 2. Methods

### 2.1. Patients and Study Design

We retrospectively reviewed 467 mNGS results in the Second Xiangya Hospital of Central South University from November 2017 to June 2019. The main sample types of mNGS in our patients were blood (324, 69.4%) and BALF (94, 20.1%), while other sample types included cerebrospinal fluid, pleural fluid, peritoneal fluid, urine, sputum, and so on (data not shown). Among them, both blood and BALF samples for mNGS tests were collected from 40 patients with the sampling interval less than 24 hours, which was defined as having paired blood and BALF mNGS results in our study. Finally, paired blood and BALF mNGS results in 39 patients with lung infiltration and suspected pulmonary infection were included for this study after excluding one patient whose final diagnosis was lung cancer. For bacteria and fungi, we compared the mNGS results of blood and BALF in patients with a paired BALF and/or blood culture result. As for viruses, we described the number of sequences in the mNGS results of BALF and blood and did not compare with the conventional respiratory viruses test due to the lack of conventional tests.

In our study, pathogen diagnosis was made according to the comprehensive analysis of the clinical examinations, mainly based on conventional culture results. For common contaminating organisms such as coagulase-negative *Staphylococcus*, viridans group streptococci, *Bacillus* species, *Corynebacterium* species, and *Propionibacterium* species, one time of single-bottle blood culture positive result was considered negative in this study [19, 20]. Besides, RNA viruses were not included for analysis because mNGS only sequenced DNA in this study.

### 2.2. Sample Processing and Sequencing

3-4 mL of blood was drawn from patients, placed in EDTA tubes, and stored at room temperature for 3–5 minutes before plasma separation and centrifuged at 1,600 g for 10 min at 4°C within 8 hours of collection. Plasma samples were transferred to new sterile tubes. DNA was extracted from 300 *μ*L of plasma using the TIANamp Micro DNA Kit (DP316, TIANGEN BIOTECH, Beijing, China) following the manufacturer's operational manual. The extracted DNA specimens were used for the construction of DNA libraries [21].

BALF was collected based on the standard clinical procedure. Briefly, 20 ml saline was injected into a segmental bronchus and drew back after a while. 3 ml of BALF was inactivated at 65°C for 30 minutes immediately after collection. 1.5 mL microcentrifuge tube with 0.5 mL sample and 1 g 0.5 mm glass beads were attached to a horizontal platform on a vortex mixer and agitated vigorously at 2800–3200 RPM for 30 min. 0.3 mL sample was separated into a new 1.5 mL microcentrifuge tube and DNA was extracted using the TIANamp Micro DNA Kit (DP316, TIANGEN BIOTECH) according to the manufacturer's recommendation.

According to the protocol of the BGISEQ-50 sequencing platform, the DNA library was constructed through DNA-fragmentation, end-repair, adapter-ligation, and PCR amplification. The constructed library was qualified by Agilent 2100 (Agilent Technologies, Santa Clara, CA) and Qubit 2.0 (Invitrogen, USA). The qualified double-strand DNA library was transformed into a single-stranded circular DNA library through DNA-denaturation and circularization. DNA nanoballs (DNBs) were generated from single-stranded circular DNA using rolling circle amplification (RCA). The DNBs were qualified using Qubit 2.0. Qualified DNBs were loaded into the flow cell and sequenced (50 bp, single-end) on the BGISEQ-50 platform.

### 2.3. Bioinformatic Analysis

High-quality sequencing data were generated by removing low-quality and short (length < 35 bp) reads using in-house software, followed by computational subtraction of human host sequences mapped to the human reference genome (hg19) using Burrows–Wheeler Alignment [22]. After the removal of low-complexity reads, the remaining data were classified by simultaneously aligning to four Microbial Genome Databases, consisting of viruses, bacteria, fungi, and parasites. The four Microbial Genome Databases were downloaded from NCBI (ftp://ftp.ncbi.nlm.nih.gov/genomes/). RefSeq contains 4,061 whole-genome sequences of viral taxa, 2,473 bacterial genomes or scaffolds, 199 fungi, and 135 parasites associated with human diseases. The number of unique alignment reads was calculated and standardized to get the number of reads stringently mapped to pathogen species (SDSMRN) and the number of reads stringently mapped to pathogen genus (SDSMRNG). The amount of sequencing data produced by BALF and blood mNGS after each step was shown in Figure S1.

### 2.4. Threshold Criteria for Interpretation of Metagenomic Analysis

The microbial list obtained from the above analysis process was compared with an in-house background database, which contains microorganisms appearing in more than 50% samples in the laboratory in the past three months. The suspected background microorganisms were removed from the microbial list. Compared with the negative control group, microorganisms with SDSMRN > 50 and at least 3 times higher than that in controls were considered as suspected pathogens, while the suspected pathogens with SDSMRN < 50 should have SDSMRN at least 5 times higher than controls.

For different types of microbes, the thresholds were set as follows:

1. Bacterium/mycoplasma/chlamydia: SDSMRNG ≥ 3
2. DNA virus/fungus: SDSMRN ≥ 3
3. Parasite: SDSMRN ≥ 100
4. *Mycobacterium tuberculosis* complex (MTC): SDSMRNG ≥ 1

To discriminate the infection from contamination, all microbes filtered by the above rules were indexed against a table of established pathogens derived from the literature and clinical guidelines according to a previous study by Langelier et al. (Table S1) [6]. Microbes were identified as putative pathogens if they were listed in the table.

### 2.5. Statistical Analysis

Continuous variables were expressed as the median with interquartile ranges (IQRs). A 2 × 2 contingency table was used to determine the sensitivity and specificity between mNGS and culture-based diagnosis in blood and BALF samples for patients with paired culture results, respectively. NcNemar test and Wilcoxon test were used in the comparative analysis for paired samples. All tests were two-sided. *p* value ≤ 0.05 was considered statistically significant. All statistical analyses were conducted using IBM SPSS Statistics for Windows, Version 25.0. Armonk, NY: IBM Corp.

### 2.6. Ethics Consideration

This study was approved by the institutional review board of the Second Xiangya Hospital (No. luoh201906).

## 3. Results

### 3.1. Baseline Characteristics

Between November 2017 and June 2019, 39 patients with lung infiltration and suspected pulmonary infection were included in this study. Demographic characteristics and clinical symptoms are shown in Table 1. The median age of these 39 patients (76.9% were male) was 56 years and the majority were above 40 years old (74.4%). The most common symptoms were cough (76.9%), fever (66.7%), and dyspnea (53.8%), followed by hemoptysis (7.7%). 14 patients had an immunocompromised history, the basic medical conditions of which included solid organ transplantation, malignancy, chemotherapy, immunosuppression for chronic kidney disease, and rheumatological diseases. Only 21 patients (53.8%) had elevated white blood cell count and 32 patients (82.1%) had elevated neutrophil ratio. The medium value of inflammatory biomarkers (procalcitonin, C-reactive protein, and erythrocyte sedimentation rate) was higher than the normal range. The time from disease-onset to sampling ranged from 0 to 90 days with a median of 22 days. 35 patients (89.3%) were admitted into the intensive care unit, while the other 4 patients were hospitalized in the emergency room. The median values of APACHE00490049 and SOFA score were 14.0 and 4.5. The median days in hospital and in ICU were 16 and 12 days.

### 3.2. Pathogens Identified by Bacteria and Fungi Cultures

Although almost all patients (38/39) had prior antibiotic treatment before sampling, BALF bacteria and fungi culture detected pathogens in 16 patients (41.0%), while blood culture was positive in only one patient whose final pathogen diagnosis was *Ralstonia pickettii*. In five patients, more than one pathogen was detected by culture simultaneously. These codetected pathogens included *Acinetobacter baumannii*, *Mycobacterium tuberculosis*, *Stenotrophomonas maltophilia*, *Candida albicans*, *Candida parapsilosis*, *Aspergillus flavus*, and *Pseudomonas aeruginosa*. Other pathogens identified by culture were *Klebsiella pneumoniae*, *Aspergillus fumigatus*, *Burkholderia cepacia*, and *Haemophilus influenzae* (Table S2).

### 3.3. Blood and BALF mNGS for Bacteria and Fungi Pathogen Identification

**Figure 1:**
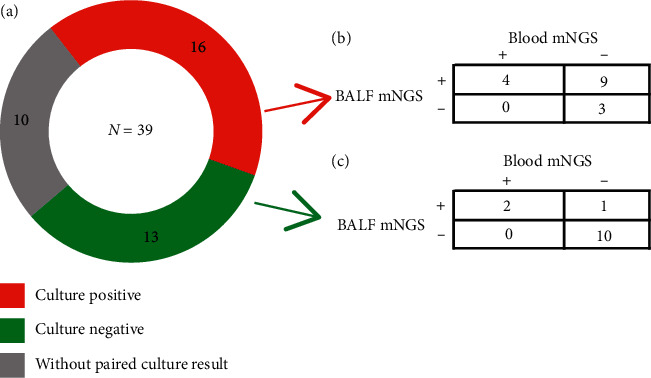
Blood and BALF mNGS for bacteria and fungi pathogen identification. (a) The culture results in 39 patients. 16 patients had positive culture results, shown in red; 13 patients had negative results, shown in green; the remaining 10 patients had no paired culture results which were sampled simultaneously with mNGS, shown in grey. (b) Pathogen identification of BALF and blood mNGS in patients with positive culture results. The sensitivity of the BALF mNGS and blood mNGS was (13/16 = 81.3%) and (4/16 = 25.0%), respectively. (c) Pathogen identification of BALF and Blood mNGS in patients with negative culture results. The specificity of the BALF mNGS and blood mNGS was (10/13 = 76.9%) and (11/13 = 84.6%), respectively. Abbreviations: mNGS, metagenomic next-generation sequencing; BALF, bronchoalveolar lavage fluid.

In all 39 patients, 16 patients had paired positive culture results and 13 patients had negative culture results, while for the other 10 patients no paired culture results were available (Figure 1 (a)). The sensitivity of the BALF mNGS (13/16 = 81.3%) was significantly (*p*=0.003) greater than that of blood mNGS (4/16 = 25.0%) (Figure 1 (b)). No difference in specificity between BALF mNGS (10/13 = 76.9%) and blood mNGS (11/13 = 84.6%) was observed (*p*=0.317) (Figure 1 (c)).

In the 16 patients who had culture-based pathogen diagnoses, blood and BALF mNGS detected the same pathogens as culture in four patients, and the number of reads in blood mNGS was smaller than BALF mNGS in all these patients. BALF mNGS was negative or detected other pathogens in the following three patients. Patient No. 21, who had a history of heart-lung transplantation, was diagnosed as *Ralstonia pickettii* infection based on the BALF culture result and clinical manifestations, while the BALF mNGS only detected *Enterococcus faecium*. Patient No. 31, whose BALF culture showed *Pseudomonas aeruginosa* infection, while both BALF mNGS and blood mNGS detected *Klebsiella pneumoniae* and *Pneumocystis jirovecii* rather than *Pseudomonas aeruginosa*, was diagnosed as *Pneumocystis jirovecii* Pneumonia according to the analysis of the clinical and radiological data. Patient No. 14 was diagnosed as *Burkholderia cepacian* infection; however, the BALF mNGS showed the most abundant bacterium was *Enterococcus faecium* (Table S2).

In the 13 patients whose culture results yielded a negative result for bacteria and fungi, BALF mNGS was positive in 3 patients, and two of them also had positive blood mNGS results. Patient No. 35 was identified as *Pneumocystis jirovecii* infection by both BALF and blood mNGS, and trimethoprim/sulfamethoxazole therapy was continued. However, BALF and blood mNGS results in patients No. 24 and No. 17 were not consistent. For patient No. 24, BALF mNGS detected 3 reads of *Pseudomonas aeruginosa* while blood mNGS result was negative. For patient No. 17, BALF mNGS identified 17 reads of *Enterococcus faecium*, but blood mNGS only detected 7 reads of *Candida parapsilosis*.

In the 10 patients without culture results, clinical treatment was changed in two patients (patient No. 03, patient No. 15) according to the BALF and blood mNGS results (Table 2). Two patients (patient No. 06 and patient No. 33) were diagnosed as *Pneumocystis jirovecii* Pneumonia and the trimethoprim/sulfamethoxazole treatment was continued. For patient No. 33, both BALF and blood mNGS detected *Pneumocystis jirovecii*, and the number of sequences detected by BALF mNGS (SDSMRN = 123887) was much higher than blood mNGS (SDSMRN = 417).

### 3.4. Blood and BALF mNGS for Viral Detection

**Figure 2:**
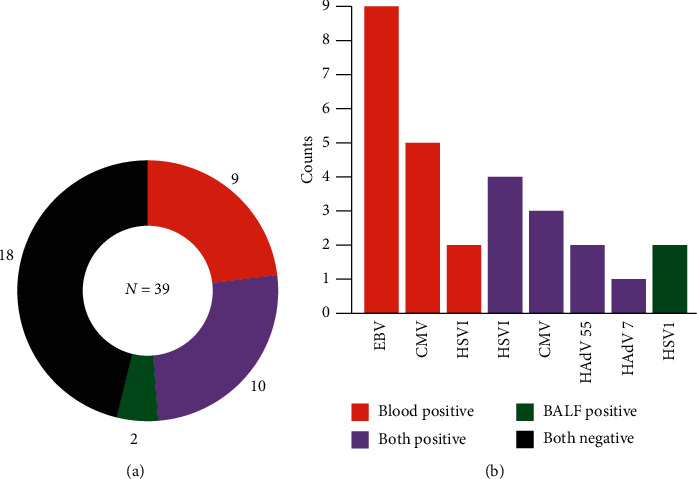
Blood and BALF mNGS for viral detection. (a) The mNGS results for viral detection in 39 patients. 18 patients (black) had negative results in both blood and BALF mNGS. Viruses were detected in 21 patients, among whom 10 patients (purple) had positive results in both blood and BALF mNGS, 9 patients (red) had positive blood mNGS results, and 2 patients (green) had positive BALF mNGS results. The number of cases and percentage of different conditions are listed near the plot. (b) The distribution of pathogens identified by blood mNGS and BALF mNGS. Abbreviations: mNGS, metagenomic next-generation sequencing; BALF, bronchoalveolar lavage fluid; EBV, Epstein-Barr virus; CMV, *Cytomegalovirus*; HAdV, human adenovirus.

**Figure 3:**
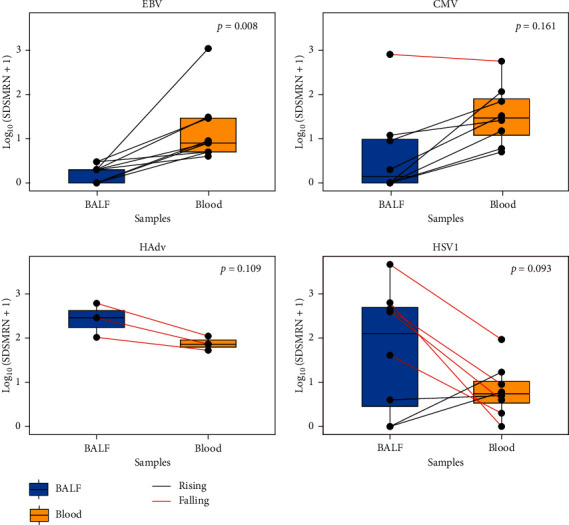
Comparison of the number of reads in BALF and blood mNGS for different viruses. The Log_10_(SDSMRN + 1) of viruses detected by BALF and blood mNGS were shown in blue and yellow. The paired data were connected by a straight line: red suggested a higher result in BALF and black suggested higher in blood. Abbreviations: mNGS, metagenomic next-generation sequencing; BALF, bronchoalveolar lavage fluid; EBV, Epstein-Barr virus; CMV, *Cytomegalovirus*; HAdv, human adenovirus; HSV1, Herpes Simplex Virus type 1; SDSMRN, the number of reads stringently mapped to pathogen species.

MNGS detected Epstein-Barr virus (EBV), *Cytomegalovirus* (CMV), human adenovirus (HAdV) type 55 and type 7, and Herpes Simplex Virus type 1 (HSV-1) in 39 patients (Figure 2). Nine patients showed positive results for viruses only in blood mNGS, two patients showed positive results only in BALF mNGS, and ten patients had positive results in both blood and BALF mNGS (Figure 2(a)). Blood mNGS detected EBV in 9 patients (Figure 2(b)), the number of sequences ranging from 3 to 1094, while all BALF mNGS results in these 9 patients were negative for EBV. In three patients whose final diagnoses were adenovirus pneumonia, both BALF and blood mNGS identified human adenovirus, while BALF mNGS detected more sequences than blood mNGS in all three patients. Eight patients showed positive results in BALF or blood mNGS for CMV. The sequences number of CMV in blood was higher than BALF except in one patient who just received heart-lung transplantation for 21 days. BALF mNGS detected more sequences than blood mNGS in five patients, but blood mNGS identified more sequences in the other three patients (Figure 3).

## 4. Discussion

We found that blood and BALF performed differently as mNGS samples in pathogen detection of pneumonia. BALF mNGS was more sensitive than blood mNGS for bacterial and fungal detection (81.3% vs. 25.0%, *p*=0.003), while no significant difference in specificity between BALF mNGS and blood mNGS was observed (76.9% vs 84.6%, *p*=0.317). However, blood mNGS detected more viruses than BALF mNGS overall, though in some patients the BALF mNGS detected more sequences for one specific virus than blood. The different viral abundance in blood and BALF samples may indicate diverse infectious status in the lung and other sites such as blood [23].

Currently, there is no previous study comparing the diagnostic performance of mNGS in blood and BALF for pneumonia. Our data indicated that BALF mNGS was more sensitive than blood mNGS for bacterial and fungal pulmonary infections, which was consistent with research about the conventional test that the yield of BALF culture was higher than that of blood culture [12]. For viruses, blood mNGS generally detected more viruses than BALF mNGS. However, for some viruses such as EBV and CMV, if they were detected by blood mNGS but were negative in BALF mNGS, it might be possible that the detected virus was not an etiological pathogen for pulmonary infection, because some viruses may shed from other body sites rather than lung or reactivated from the blood [23, 24]. In patients whose final clinical diagnosis was viral pneumonia, the BALF mNGS detected more sequences than blood in our study, which was consistent with previous studies that showed a higher level of CMV DNA detected in BALF compared with the blood using real-time PCR [25, 26]. Pathogen DNA in blood may also reflect the pulmonary infection because high blood flow in the lung may lead to increased pathogen DNA shedding [18].

In the 16 patients whose diagnoses were confirmed by culture, mNGS detected additional microbes including *Enterococcus faecium*, *Pneumocystis jirovecii*, *Acinetobacter baumannii*, *Streptococcus pneumoniae*, *Candida glabrata*, *Klebsiella pneumoniae*, *Haemophilus influenzae*, *Candida parapsilosis*, and *Aspergillus fumigatus*. *Enterococcus faecium* was detected in three patients, two of whom were immunosuppressed and the blood and BALF mNGS were both positive for *Enterococcus faecium*. For another patient, *Enterococcus faecium* was only detected by BALF, and *Enterococcus faecium* was not interpreted as an etiological pathogen because *Enterococcus faecium* pneumonia was uncommon in an immunocompetent patient [27]. For one patient who had chronic kidney disease, *Pneumocystis jirovecii* was detected by BALF mNGS, *Klebsiella pneumoniae* was detected by both BALF and blood mNGS, and the (1,3)-beta-D-glucan test and galactomannan (GM) test was positive. Although other specific tests for *Pneumocystis jirovecii* such as culture, PCR, and methenamine-silver stain were not available, this patient was diagnosed as *Pneumocystis jirovecii*, *Klebsiella pneumoniae*, and *Pseudomonas aeruginosa* mixed infection according to the culture result, clinical performance, and radiological data. Other microbes detected by mNGS were not regarded as pathogens because of the lack of additional supportive culture results or clinical characteristics.

False-negative occurred in three patients for BALF mNGS. The culture result of patient No. 14 showed the pathogen was *Burkholderia cepacia*, but both BALF and blood mNGS only detected *Enterococcus faecium*, which may be attributed to inadequate sequences captured by mNGS that specifically aligned to the genome of *Burkholderia cepacia*. BALF and blood culture showed patient No. 21 was infected with *Ralstonia pickettii* which was not in the report list of BALF and blood mNGS. In the raw result of BALF mNGS, *Ralstonia pickettii* has the highest number of reads (SDSMRN = 101) in BALF mNGS but was filtered as suspected background microorganisms. Patient No. 31 was diagnosed as a mixed infection with *Pneumocystis jirovecii*, *Klebsiella pneumoniae*, and *Pseudomonas aeruginosa*. BALF and blood mNGS did not detect *Pseudomonas aeruginosa* which was positive in BALF culture. The false-positive result may relate to the high proportion of host sequences and inadequate sequencing depth for the microbiomes [2].

Blood mNGS detected more viruses than BALF in our patients. As for EBV, blood mNGS was positive in 8 patients whose BALF mNGS results were all negative according to our positive criteria for viruses. This result was consistent with the fact that few reports of pneumonia were attributable to EBV infection [28], which indicated that EBV detected only by blood mNGS might not relate to the pulmonary infection, but were more likely to be considered as a complication of impaired host immunity. For human adenovirus, both blood and BALF were positive for 3 patients whose final diagnosis was adenovirus pneumonia. Although blood mNGS detected lower sequences than BALF mNGS, the number of sequences in the blood was adequate to make a diagnosis. For CMV and HSV1, it seems that BALF mNGS detected fewer sequences for CMV and more sequences for HSV1 than blood mNGS. However, BALF mNGS detected more sequences in one patient who just received heart-lung transplantation for 21 days. BALF mNGS results were negative in three patients whose blood mNGS results were positive for HSV1. Blood mNGS detected more viruses than BALF overall, which signified that viruses were more likely to shed into blood compared with bacteria or fungi. However, the positive blood mNGS result for viruses might also indicate viruses reactivated from other organs or blood [23].

Previous studies have shown great concordance between mNGS and conventional microbiologic testing [6, 13, 14, 29, 30] and suggested mNGS might lead to better clinical prognosis than conventional testing in severe pneumonia in intensive care unit [13]. Our study also showed BALF mNGS had good concordance with culture results (sensitivity = 81.3%, specificity = 76.9%), while there were also some inconsistencies between blood mNGS and BALF mNGS as well as between conventional tests and mNGS. Factors that may account for false negative and false positive mNGS results include the following: (a) inadequate sequencing depth; (b) prior antibiotic usage; (c) high host genome background and low microbial biomass of the true pathogens; (d) strict filtering strategy for mNGS results; (e) contamination of microbial genome from the environment or body flora.

Our study has some limitations. First, the sample size was small. Considering that the patients' situation could change very quickly in the hospital, the mNGS results for one patient may be incomparable if the sampling interval between blood and BALF was too long. To analyze the consistency of paired blood and BALF samples, we limited our inclusion criteria as the sampling interval should be less than 24 hours for one patient, which excluded some patients whose blood and BALF samples for mNGS were collected in a different day. Second, we used the culture-based result as the reference standard to evaluate the diagnostic performance of mNGS, which might underestimate the sensitivity of mNGS because false-positive could exist in culture. Meanwhile, many other clinical auxiliary inspections such as sputum Gram staining, serum immunological test, *β*-d-glucan test, galactomannan antigen test, and pathogen microarray were not included in the conventional test in our study, resulting in loss of useful information which otherwise might be helpful to pathogen diagnosis in pulmonary infection. Only DNA was sequenced in our study, which means all RNA viruses were excluded such as influenza. Furthermore, considering the variable periods from disease onset to sampling in our patients, the mNGS result or conventional test result not only might reflect the original pathogen responsible for community-acquired pneumonia but also could be pathogens related to hospital-acquired pneumonia or even co-infection in some patients, which made it difficult to interpret all the microbiological results. Finally, almost all patients in this study were given prior antimicrobic treatment before sampling, which might decrease the positive rate of the conventional test as well as the sensitivity of mNGS.

As a revolutionary diagnostic tool, mNGS can detect all pathogens simultaneously. However, some disadvantages are inherent to mNGS. For example, one is the microbial contaminant, which may complicate the interpretation of mNGS results and lead to unnecessary tests and inappropriate treatment; another is high human host background, which may limit the yield of pathogen sequences, thus accounting for the inadequate sensitivity of mNGS [1]. Along with other disadvantages like relatively high cost, these weaknesses considerably limited the clinical application of mNGS in the field of pathogen identification. Development of new host depletion methods [31], research of body flora composition [32], and the evolution of new sequencing technologies may address these questions in the near future.

## 5. Conclusion

Together, both blood and BALF mNGS can aid the identification of pathogens for pulmonary infections. Our study suggests BALF mNGS is superior in detecting bacterial and fungi pathogens, while blood mNGS has advantages for viral infection surveillance, which we hope may help clinicians to make clinical decisions about whether to conduct mNGS for pathogen diagnosis in pneumonia and what is the better sample to choose.

## Figures and Tables

**Table 1:** Baseline characteristics of 39 patients.

Characteristic	Value (median (IQRs) or no. (%))
Age (years)	
Years	56 (34, 66)
Distribution	
≤18 years old	4 (10.3%)
19–40 years old	6 (15.3%)
41–60 years old	12 (30.8%)
>60 years old	17 (43.6%)
Male sex	30 (76.9%)
Onset symptoms	
Dyspnea	21 (53.8%)
Fever	26 (66.7%)
Cough	30 (76.9%)
Hemoptysis	3 (7.7%)
Inflammation biomarker	
WBC (10^9^/L)	10.5 (8.5, 14.8)
NEU (%)	85.8 (77.7, 90.3)
Lym (10^9^/L)	0.78 (0.59, 1.47)
PCT (ng/ml)	0.81 (0.17, 2.3)
CRP (mg/L)	122 (34, 187)
ESR (mm/h)	57 (29, 84)
Comorbidity	
Diabetes mellitus	7 (17.9%)
Chronic kidney disease	9 (23.1%)
Liver disease	4 (10.3%)
Immunocompromised	
Solid organ transplantation	2 (5.1%)
Malignancies	1 (2.6%)
Chemotherapy	3 (7.7%)
Immunosuppression for rheumatological diseases	4 (10.3%)
Immunosuppression for chronic kidney disease	4 (10.3%)
ICU admission	35 (89.7%)
Severity score	
APACHE II	14.0 (7.8, 21.5)
SOFA	4.5 (2.0, 9.0)
Disease-onset to sampling time (days)	22 (12, 36)
Length of stay (days)	
In hospital	16 (8, 39)
In ICU	12 (4, 30)

Abbreviations: WBC, white blood cells; NEU%, percentage of neutrophil; Lym, lymphocytes; PCT, procalcitonin; CRP, C reactive protein; ESR, erythrocyte sedimentation rate; APACHE, acute physiology and chronic health evaluation scoring system; SOFA, sequential organ failure assessment; ICU, intensive care unit.

**Table 2:** The mNGS results of bacteria and fungi for patients without paired BALF culture result (*n* = 10).

Patient ID	BALF mNGS result	SMRN	Blood mNGS result	SMRN	Clinical diagnosis	Changes in treatment
03	*Stenotrophomonas maltophilia*	14	—	—	HAP	Discontinuation of Tigecycline
	*Candida albicans*	6				
05	*Candida tropicalis*	5	—	—	HAP	No change
06	—	—	*Pneumocystis jirovecii*	40	PJP	No change
15	—	—	—	—	CAP	Discontinuation of Amphotericin B
30	*Candida tropicalis*	49	—	—	HAP	No change
	*Candida glabrata*	4				
33	*Pneumocystis jirovecii*	123887	*Pneumocystis jirovecii*	417	PJP Bacterial infection	No change
	*Acinetobacter baumannii*	553				
	*Escherichia coli*	27				
36	—	—	—		CAP	No change
37	—	—	*Acinetobacter baumannii*	6	CAP	No change
38	—	—	—	—	HAP	No change
39	—	—	—	—	Aspiration pneumonia	No change

Abbreviations: mNGS, metagenomic next-generation sequencing; SMRN, number of reads stringently mapped to pathogen species; PJP, *Pneumocystis jirovecii* Pneumonia; CAP, community-acquired pneumonia; HAP, hospital-acquired pneumonia.

## Data Availability

The datasets analyzed during the current study are available from the corresponding author upon reasonable request.

## Supplementary Materials

Supplementary Materials Figure S1: the amount of sequencing data produced by BALF and blood mNGS. Table S1: reference index of established lower respiratory tract pathogens. Table S2: BALF and blood mNGS and culture results for bacteria and fungi of 39 patients.
