# Supplementary material for: Blood and Bronchoalveolar Lavage Fluid Metagenomic Next-Generation Sequencing in Pneumonia
Source: Can J Infect Dis Med Microbiol. 2020 Aug 12;2020:6839103. doi: 10.1155/2020/6839103 (PMC7448216; doi:10.1155/2020/6839103)
Supplement: Supplementary Materials — Figure S1: the amount of sequencing data produced by BALF and blood mNGS. Table S1: reference index of established lower respiratory tract pathogens. Table S2: BALF and blood mNGS and culture results for bacteria and fungi of 39 patients. [file 6839103.f1.zip › 6839103.f1/Fig S1.docx]

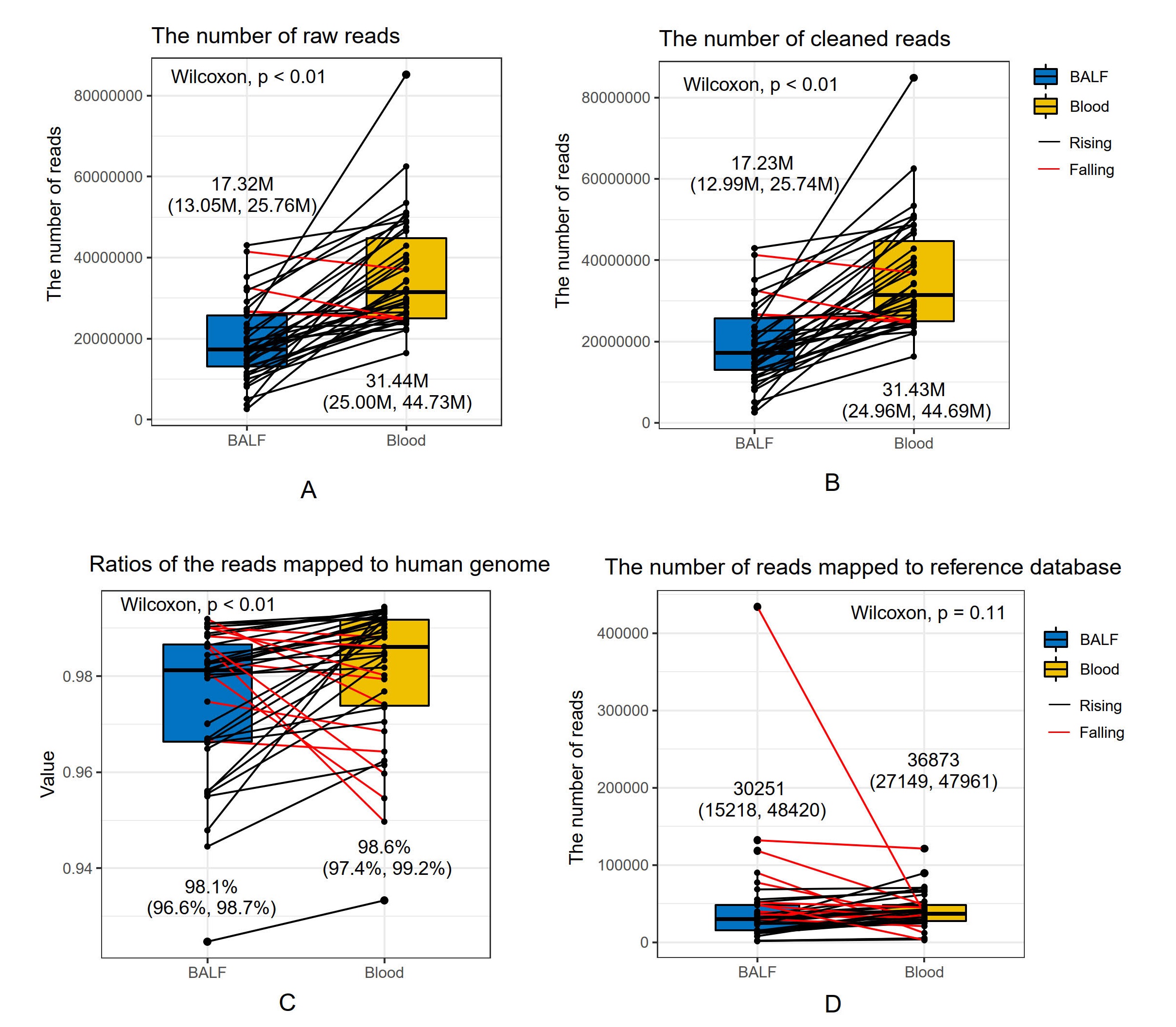


**Figure S1. The amount of sequencing data produced by BALF and blood mNGS.**

(A) MNGS yielded a median of 17.32 million and 31.44 million raw reads for BALF and blood (p<0.01). (B) The median number of cleaned reads for BALF and blood mNGS were 17.23 million and 31.43 million respectively (p<0.01). (C) Majority of the clean reads were mapped to the human genome with a median rate of 98.12% in BALF and 98.61% in blood mNGS (p<0.01). (D) No statistical difference was observed between BALF and blood mNGS for the number of reads mapped to the microorganism reference database (BALF median = 30251, interquartile range = 15218- 48420; blood median=36873, interquartile range = 27149-47961; p = 0.11).
